# Augmentation of curved tip of left-sided double-lumen tubes to reduce right bronchial misplacement: A randomized controlled trial

**DOI:** 10.1371/journal.pone.0210711

**Published:** 2019-01-15

**Authors:** Jeong-Hwa Seo, Susie Yoon, Se-Hee Min, Hyung Sang Row, Jae-Hyon Bahk

**Affiliations:** Department of Anesthesiology and Pain Medicine, Seoul National University Hospital, Seoul National University College of Medicine, Seoul, Republic of Korea; Medizinische Universitat Graz, AUSTRIA

## Abstract

**Background:**

During intubation with a blind technique, a left-sided double-lumen tube (DLT) can be misdirected into the right bronchus even though its curved tip of the bronchial lumen turns to the left. This right bronchial misplacement may be associated with the tip angle of DLTs. We thus performed a randomized trial to test the hypothesis that the DLT with an acute tip angle enters the right bronchus less frequently than the tube with an obtuse tip angle.

**Methods:**

We randomized surgical patients (n = 1427) receiving a polyvinyl chloride left-sided DLT. Before intubation, the curved tip was further bent to an angle of 135° and kept with a stylet inside in the curved-tip group, but not in the control group. After the tip was inserted into the glottis under direct or video laryngoscopy, the stylet was removed and the DLT was advanced into the bronchus with its tip turning to the left. We checked which bronchus was intubated, and the time and number of attempts for intubation. After surgery, we assessed airway injury, sore throat, and hoarseness. The primary outcome was the incidence of right bronchial misplacement of the DLT.

**Results:**

DLTs were misdirected into the right bronchus more frequently in the control group than in the curved-tip group: 57/715 (8.0%) vs 17/712 (2.4%), risk ratio (95% CI) 3.3 (2.0–5.7), P < 0.001. The difference was significant in the use of 32 (P = 0.003), 35 (P = 0.007), and 37 (P = 0.012) Fr DLTs. Intubation required longer time (P < 0.001) and more attempts (P = 0.002) in the control group. No differences were found in postoperative airway injury, sore throat and hoarseness.

**Conclusions:**

Before intubation of left-sided DLTs, augmentation of the curved DLT tip reduced the right bronchial misplacement and facilitated intubation without aggravating airway injury.

## Introduction

For lung isolation during thoracic surgery, a left-sided double-lumen endobronchial tube (DLT) is used more commonly than a right-sided one because the left mainstem bronchus is longer than the right mainstem bronchus and has a greater margin of safety for correct positioning of the DLT. [1] After the left-sided DLT passes the glottis and its curved tip of the bronchial lumen turns to the left side, it is advanced into the left bronchus either blindly or by fiberoptic bronchoscopic guidance. [2–5] Although using the bronchoscopic guiding method would improve the safety of the maneuver, using the blind technique is easier and faster to place the DLT into the left bronchus. [6] However, when the blind technique is applied, the tube may be misdirected into the right bronchus because the right bronchus is wider and more vertical than the left bronchus. [7–12] If the DLT is misplaced into the right bronchus, this may inhibit ventilation or deflation of the right upper lobe and thus worsen arterial oxygenation or surgical view. [1, 13, 14] Moreover, the bronchoscopic guidance of the DLT from the right to the left bronchus is technically difficult, and the bronchoscope may be broken during the manipulation. [15] Therefore, it is important to prevent the right bronchial misplacement during blind intubation of left-sided DLTs.

A previous retrospective study suggested that the left-sided DLT with an obtuse tip angle was more likely to enter the right bronchus probably because the curved tip may not reach the left bronchial opening even after left rotation of the DLT. [16] We thus performed a prospective randomized trial comparing the right bronchial misplacement of left-sided DLTs with different tip angles. Our hypothesis was that the DLT with an acute tip angle enters the right bronchus less frequently than the tube with an obtuse tip angle.

## Materials and methods

### Patients

This prospective, single-center, parallel-group, double-blind, randomized controlled trial was approved by the Institutional Review Board of Seoul National University Hospital (1301-040-457) and registered at ClinicalTrials.gov (NCT01818128). Written informed consent was obtained from each patient prior to the study. We enrolled patients aged 20–85 years with American Society of Anesthesiologists physical status of I–III, and undergoing elective surgery using polyvinyl chloride (PVC) left-sided DLTs (Mallinckrodt endobronchial tube; Covidien, Mansfield, MA, USA) between March 2013 and February 2015. We excluded patients with tracheobronchial abnormalities, cervical spine diseases, loose teeth, interincisor distance of < 3 cm, thyromental distance of < 6 cm, neck range of motion of < 90°, Mallampati class of ≥ 3, and body mass index of ≥ 35 kg m^-2^.

**Fig 1:**
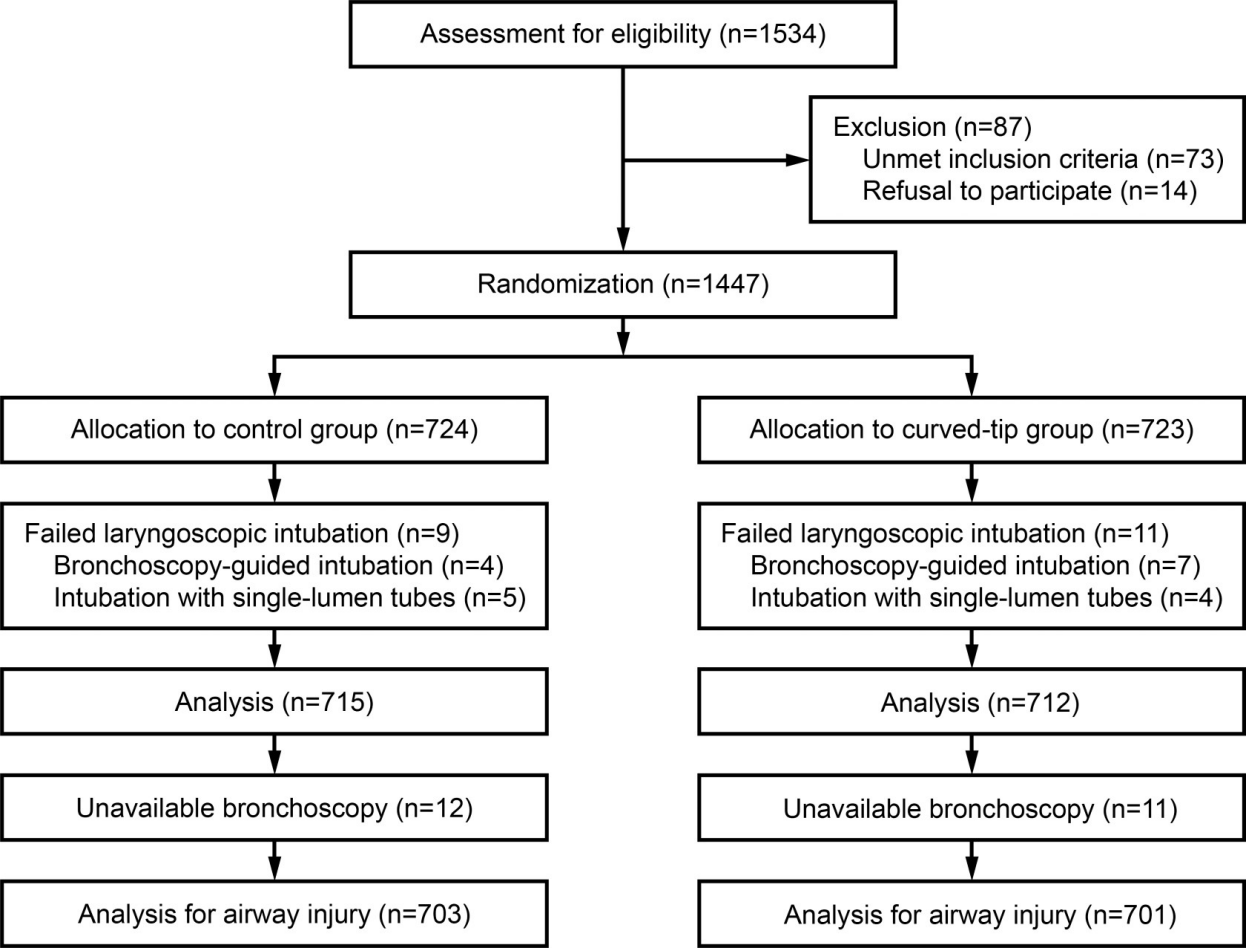
CONSORT flow diagram.

Patients were randomized into two groups depending on whether the DLT tip was further bent or not before intubation (Fig 1). A statistician not involved in the trial created a randomization in a 1:1 ratio using random block sizes of 2, 4, 6, 8, and 10. The allocation sequence was concealed in sequentially numbered, opaque, and sealed envelopes. Patients and investigators were blinded to group assignment.

### Preparation of double-lumen tubes

Two investigators (SY, S-HM) measured the inner diameters of the left and right mainstem bronchi on the preoperative computed tomography. [16–19] The DLT size was selected based on the left bronchial diameter or sex and height of the patient. [4, 5, 16, 19]

**Fig 2:**
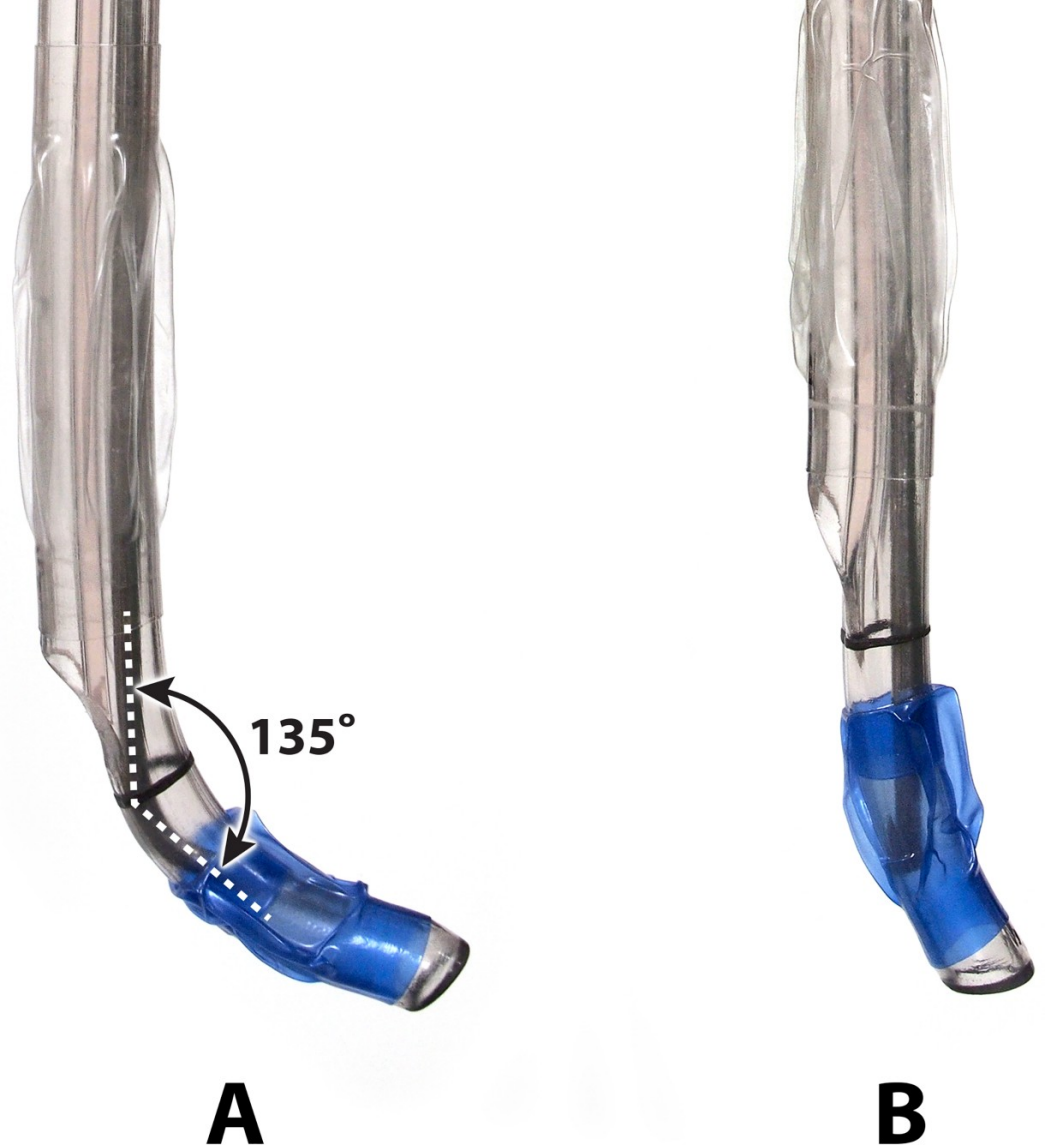
A 37-Fr double-lumen tube with its bronchial tip bent to an angle of 135° (A) and a tube without any modification to the tip (B).

Nurses unaware of the study protocol prepared the DLT in an aseptic manner. The DLT had a stylet inside the bronchial lumen, and the tracheal and bronchial cuffs were deflated but not lubricated. At the midpoint between the proximal margin of the bronchial cuff and the radiopaque line, the curved tip was further bent to an angle of 135° by using a digital protractor (iGaging, San Clemente, CA, USA) in the curved-tip group, but not in the control group (Fig 2).

### Anesthesia and intubation

The investigators (J-HS, SY, S-HM) instructed the attending anesthetists on how to conduct the intubation of left-sided DLTs, but were out of the operating room during anesthetic induction. Patients were monitored with blood pressure, electrocardiograph, pulse oximeter, bispectral index (A-2000 XP; Aspect Medical Systems, Newton, MA, USA), and acceleromyograph (TOF-watch SX; Organon, Dublin, Ireland). A headrest of 9-cm height was placed under the patient’s occiput.

Anesthesia was induced with effect-site target-controlled infusion (Orchestra; Fresenius Kabi, Brézins, France) of propofol and remifentanil. The initial effect-site concentrations were 3–5 ug ml^-1^ for propofol and 3–5 ng ml^-1^ for remifentanil. Rocuronium 0.6–0.8 mg kg^-1^ was administered and train-of-four (TOF) counts were monitored at the adductor pollicis muscle.

At a TOF count of 0 and a bispectral index of < 60, the attending anesthetist performed intubation under direct laryngoscopy using a Macintosh blade of 3 or 4. The bronchial tip was inserted into the glottis directing anteriorly and the stylet was removed. The DLT was rotated 90° counterclockwise turning the tip towards the left and advanced to the pre-estimated depth, [4, 5, 20] and then the laryngoscopic blade was removed from the mouth. The intubation time was defined as the interval between insertion and removal of the laryngoscopic blade in the mouth. [5] The anesthetist recorded the Cormack-Lehane grade [21] and number of intubation attempts.

If the DLT did not pass the glottis because of severe resistance, the DLT was not removed and its advancement was retried with the tube further rotated up to 180° counterclockwise. [4] If the DLT was too large to enter the trachea or bronchus, the next smaller tube was used. Video laryngoscopy (UESCOPE; UM Medical Devices, Newton, MA, USA) was applied after two failures of direct laryngoscopy. The DLT curvature was modified at the discretion of the attending anesthetist to facilitate video laryngoscopy, but the tip angle of the DLT was maintained in both groups. If both direct and video laryngoscopy failed, intubation was performed with bronchoscopic guidance and it was excluded from the analysis.

Using a fiberoptic bronchoscope (LF-DP or LF-GP; Olympus Optical Co., Tokyo, Japan), the investigator (S-HM, J-HB), who was blinded to group assignment, checked which bronchus was intubated. If the DLT entered the right bronchus, it was withdrawn back to the trachea and guided into the left bronchus with the bronchoscope. The bronchial cuff was placed in the left mainstem bronchus below the carina without herniation, and the bronchial tip was above the left lobar bronchi without obstruction. [17–19, 22–24] It was re-checked after positional change of the patient. The tracheal and bronchial cuff pressures were adjusted to less than 25 cmH_2_O with a cuff pressure monitor (VBM Medizintechnik GmbH, Sulz am Neckar, Germany).

One lung was ventilated with a tidal volume of 4–8 ml kg^-1^, PEEP of 4–8 cmH_2_O, respiratory rate of 12–20 min^-1^, and inspired oxygen fraction of 0.6–1.0. The effect-site concentrations of propofol and remifentanil were titrated within a bispectral index of 30–60, and rocuronium 0.2–0.4 mg kg^-1^ was given at a TOF count of ≥ 1. At skin closure, the patient received either intravenous or epidural patient-controlled analgesia with fentanyl, morphine, or levobupivacaine. [4, 5]

### Postoperative airway injuries, sore throat, and hoarseness

After surgery, the patient was moved to the supine position and secretions were suctioned from both lungs. After pulling the DLT above the carina, the investigator (J-HS) who was blinded to group assignment inserted the fiberoptic bronchoscope into the tube and examined the trachea, carina, and both bronchi. Sugammadex 2–4 mg kg^-1^ was given and the trachea was extubated at a TOF ratio of > 0.9. After spraying topical lidocaine into the nasal and oral cavities, the investigator (J-HS) examined the vocal cords with transnasal flexible laryngoscopy. [5] The types of injuries in the vocal cords, trachea, carina, and both bronchi were classified as redness, edema, hematoma, bleeding, and others. We defined redness as the red color in the mucosa without surrounding inflammatory swelling, edema as swollen mucosa, hematoma as previous bleeding into the mucosa, and bleeding as active bleeding. [3, 25]

If mechanical ventilation was required after surgery, the trachea was re-intubated with a single-lumen tube of an inner diameter of 7.0 mm for women and 7.5 mm for men without administration of sugammadex. The patient was transferred to the post-anesthesia room or intensive care unit.

The investigator (SY, HSR) blinded to group assignment assessed sore throat and hoarseness one hour, one and two days after extubation. The severity of sore throat was graded as none, mild pain only with swallowing, moderate constant pain aggravated during swallowing, and severe pain interfering with eating and requiring analgesics. [3–5, 25] Hoarseness was defined as an acoustic quality different from the preoperative voice and graded as follows: none; mild, noticed by the patient; moderate, obvious to the observer; and severe, aphonia. [3–5, 25]

The primary outcome was the incidence of the right bronchial misplacement of left-sided DLTs. The secondary outcomes were the time and number of attempts for intubation; and airway injuries, sore throat, and hoarseness after surgery.

### Statistical analysis

In a previous study, the incidence of the right bronchial misplacement of left-sided DLTs was 4.2%. [16] In order to have 80% power to detect a 60% decrease of the incidence in the curved-tip group using a two-tailed Fisher’s exact test at the 0.05 level of statistical significance, 705 patients per group were needed.

All continuous variables were compatible with a normal distribution using the Shapiro-Wilk test, and thus were summarised using mean and standard deviation. Categorical variables were summarized using counts and percentages. Group differences for continuous variables were assessed using unpaired t-tests. Within each group, the right and left bronchial diameters were compared using a paired t-test. Group differences for categorical variables were assessed using Fisher’s exact test. The effects of treatment on various risks were summarized using the risk ratio. The risks studied were: right bronchial misplacement, multiple intubation attempts, airway injury, sore throat, and hoarseness. The effect of treatment on intubation time was summarized using the mean difference. Statistical significance was declared at the 0.05 level. STATA (Special Edition 14.2; Stata Corporation, College Station, Texas, USA) was used for sample size calculation, randomization, and statistical analysis.

## Results

After screening 1534 patients, 715 patients were included in the control group and 712 patients in the curved-tip group (Fig 1). Reasons for exclusion were quite similar between the two groups. Twelve out of 715 patients (1.7%) in the control group and 11 out of 723 patients (1.5%) in the curved-tip group were not evaluable due to unavailable bronchoscopy.

Baseline data of patients and intubation were similar between groups (Table 1). Within each group, the right bronchial diameter was larger than the left one: Table 1; mean difference (95% CI), 1.5 mm (1.4–1.6 mm), P < 0.001 by a paired t-test in the control group; 1.6 mm (1.5–1.7 mm), P < 0.001 in the curved-tip group.

**Table 1:** Characteristics of patients, intubation, and surgery.

	Control group (n = 715)	Curved-tip group (n = 712)	P value
Age (year)	59.0 ± 14.0	57.9 ± 14.7	0.152
Gender			0.789
Female	304 (42.5%)	308 (43.3%)	
Male	411 (57.5%)	404 (56.7%)	
Weight (kg)	62.9 ± 10.7	61.9 ± 10.1	0.076
Height (cm)	162.9 ± 9.2	162.5 ± 9.3	0.487
Body mass index (kg m^-2^)	23.6 ± 3.0	23.4 ± 3.0	0.139
American Society of anesthesiologists physical status			0.740
I	258 (36.1%)	271 (38.1%)	
II	334 (46.7%)	323 (45.4%)	
III	123 (17.2%)	118 (16.6%)	
Left bronchial diameter (mm)*	11.3 ± 1.5	11.2 ± 1.4	0.195
Right bronchial diameter (mm)*	12.7 ± 1.5	12.8 ± 1.5	0.668
Anesthesiologist performing intubation			0.733
First-grade resident	101 (14.1%)	109 (15.3%)	
Second-grade resident	238 (33.3%)	224 (31.5%)	
Third-grade resident	152 (21.3%)	168 (23.6%)	
Fourth-grade resident	115 (16.1%)	105 (14.7%)	
Specialist	109 (15.2%)	106 (14.9%)	
Grade of laryngoscopic view			0.955
1	518 (72.4%)	516 (72.5%)	
2	152 (21.3%)	154 (21.6%)	
3	45 (6.3%)	42 (5.9%)	
Intubaion with 180° rotation technique	29 (4.1%)	27 (3.8%)	0.892
Use of next smaller double-lumen tube	18 (2.5%)	15 (2.1%)	0.725
Surgical position			0.940
Left lateral decubitus	404 (56.5%)	401 (56.3%)	
Right lateral decubitus	259 (36.2%)	260 (36.5%)	
Supine	47 (6.6%)	44 (6.2%)	
Other	5 (0.7%)	7 (1.9%)	
Surgical type			0.649
Thoracoscopy	583 (81.5%)	587 (82.4%)	
Thoracotomy	92 (12.9%)	88 (12.4%)	
Sternotomy	28 (3.9%)	21 (2.9%)	
Other	12 (1.7%)	16 (2.2%)	
Duration of anesthesia (min)	202.9 ± 96.1	202.1 ± 83.7	0.853
Postoperative mechanical ventilation	19 (2.7%)	12 (1.7%)	0.276
Patient-controlled analgesia			0.494
Intravenous	612 (85.6%)	609 (85.5%)	
Epidural	96 (13.4%)	91 (12.8%)	
None	7 (1.0%)	12 (1.7%)	

Values are mean ± standard deviation or number of patients (percentage). Continuous variables were compared using unpaired t-tests, and categorical variables were compared using Fisher’s exact tests.

*Measured on the preoperative computed tomography of 676 patients in the control group and 677 patients in the curved-tip group because the remaining patients did not have the examination.

DLTs were misplaced into the right bronchus more frequently in the control group than in the curved-tip group, and the differences were significant in the use of 32, 35, and 37 Fr DLTs (Table 2). One 39-Fr DLT was malpositioned in the control group (Table 2). In the comparison within each group, 32-Fr DLTs were more frequently malpositioned than 35, 37, and 39-Fr DLTs in the control group and than 37 and 39-Fr DLTs in the curved-tip group (Table 3).

**Table 2:** Between-group comparisons of the incidence of the right bronchial misplacement in the use of left-sided double-lumen tubes of different sizes.

Tube size	Control group	Curved-tip group	Risk ratio (95% CI)	P value*
32 Fr	25/128 (19.5%)	9/130 (6.9%)	2.8 (1.4–5.8)	0.003
35 Fr	19/218 (8.7%)	6/225 (2.7%)	3.3 (1.3–8.0)	0.007
37 Fr	12/274 (4.4%)	2/265 (0.8%)	5.8 (1.3–25.7)	0.012
39 Fr	1/95 (1.1%)	0/92 (0.0%)	Not applicable	> 0.999
Total	57/715 (8.0%)	17/712 (2.4%)	3.3 (2.0–5.7)	< 0.001

Values are number of patients (percentage).

*By comparing the incidence of the malposition between groups using Fisher’s exact test.

**Table 3:** Within-group comparisons of the incidence of the right bronchial misplacement between the uses of left-sided double-lumen tubes of different sizes.

	Control group			Curved-tip group		
Tube size	Incidence	Risk ratio (95% CI)	P value*	Incidence	Risk ratio (95% CI)	P value*
32 vs 35 Fr	25/128 vs 19/218 (19.5 vs 8.7%)	2.2 (1.3–3.9)	0.004	9/130 vs 6/225 (6.9 vs 2.7%)	2.6 (0.9–7.1)	0.097
32 vs 37 Fr	25/128 vs 12/274 (19.5 vs 4.4%)	4.5 (2.3–8.6)	< 0.001	9/130 vs 2/265 (6.9 vs 0.8%)	9.2 (2.0–41.8)	0.001
32 vs 39 Fr	25/128 vs 1/95 (19.5 vs 1.1%)	18.6 (2.6–134.5)	< 0.001	9/130 vs 0/92 (6.9 vs 0.0%)	Not applicable	0.011
35 vs 37 Fr	19/218 vs 12/274 (8.7 vs 4.4%)	2.0 (1.0–4.0)	0.061	6/225 vs 2/265 (2.7 vs 0.8%)	3.5 (0.7–17.3)	0.151
35 vs 39 Fr	19/218 vs 1/95 (8.7 vs 1.1%)	8.3 (1.1–61.0)	0.010	6/225 vs 0/92 (2.7 vs 0.0%)	Not applicable	0.187
37 vs 39 Fr	12/274 vs 1/95 (4.4 vs 1.1%)	4.2 (0.5–31.6)	0.197	2/265 vs 0/92 (0.8 vs 0.0%)	Not applicable	> 0.999
32 vs 35 vs 37 vs 39 Fr	25/128 vs 19/218 vs 12/274 vs 1/95 (19.5 vs 8.7 vs 4.4 vs 1.1%)		< 0.001	9/130 vs 6/225 vs 2/265 vs 0/92 (6.9 vs 2.7 vs 0.8 vs 0.0%)		0.001

Values are number of patients (percentage).

*By Fisher’s exact test.

**Fig 3:**
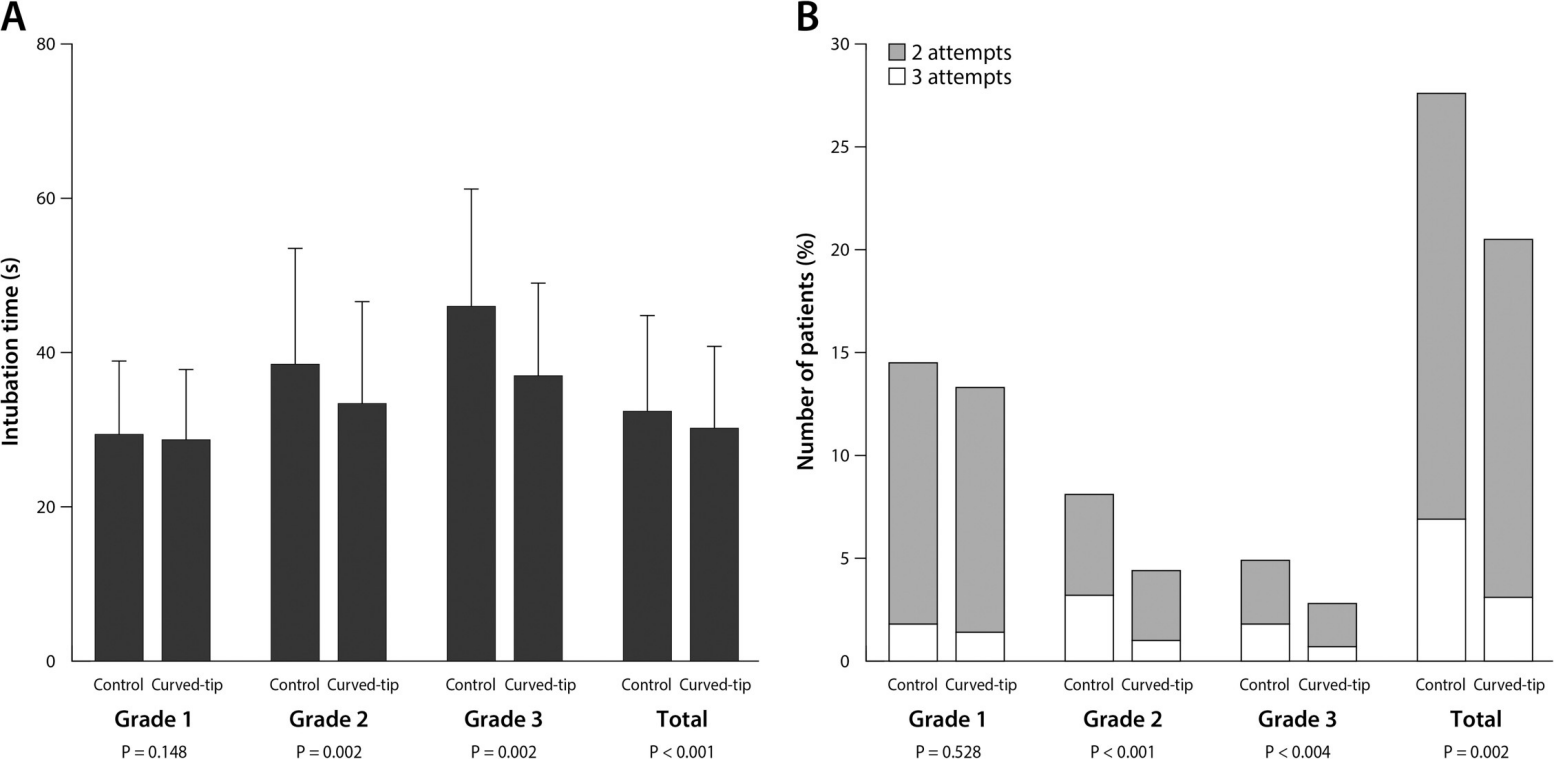
**Mean time (A) and number of attempts (B) for intubation of left-sided double-lumen tubes in patients with each grade of laryngoscopic view.** Error bars are standard deviations. P values were obtained by comparing the intubation time between groups using unpaired t-tests (A) and by comparing the number of intubation attempts using Fisher’s exact tests (B).

Intubation required longer time and more attempts in the control group than in the curved-tip group, and these differences were significant in the patients with the laryngoscopic grade of 2 and 3 (Fig 3, Table 4). Video laryngoscopy was used for the third attempt of intubation and was more frequently used in the control group than in the curved-tip group: 49/715 (6.9%) vs 22/712 (3.1%), risk ratio (95% CI) 2.2 (1.4–3.6), P = 0.001.

**Table 4:** The time and number of attempts for intubation of left-sided double-lumen tubes, the incidence and type of airway injury, and the incidence and severity of sore throat and hoarseness after surgery.

	Control group	Curved-tip group	Mean difference or risk ratio (95% CI)	P value
Intubation time (s)	32.4 ± 12.4 (n = 715)	30.1 ± 10.6 (n = 712)	2.3 (1.1–3.5)	< 0.001
Laryngoscopic grade 1	29.4 ± 9.4 (n = 518)	28.5 ± 9.1 (n = 516)	0.8 (-0.3–2.0)	0.148
Laryngoscopic grade 2	38.5 ± 15.3 (n = 152)	33.4 ± 13.2 (n = 154)	5.1 (1.9–8.3)	0.002
Laryngoscopic grade 3	46.4 ±14.6 (n = 45)	37.4 ± 11.8 (n = 42)	8.9 (3.3–14.6)	0.002
Number of intubation attempts (1/2/3)	518/148/49 (72.4/20.7/6.9%)	566/124/22 (79.5/17.4/3.1%)	1.3 (1.1–1.6)*	0.002*
Laryngoscopic grade 1	414/91/13 (57.9/12.7/1.8%)	421/85/10 (59.1/11.9/1.4%)	1.1 (0.8–1.4)*	0.528*
Laryngoscopic grade 2	94/35/23 (13.1/4.9/3.2%)	123/24/7 (17.3/3.4/1.0%)	1.9 (1.3–2.8)*	< 0.001*
Laryngoscopic grade 3	10/22/13 (1.4/3.1/1.8%)	22/15/5 (3.1/2.1/0.7%)	1.6 (1.1–2.3)*	0.004*
Vocal cord injury	253/703 (36.0%)	249/701 (35.5%)	1.0 (0.9–1.2)	0.867
Redness	147 (20.9%)	161 (23.0%)		
Edema	64 (9.1%)	59 (8.4%)		
Hematoma	35 (5.0%)	19 (2.7%)		
Bleeding	7 (1.0%)	10 (1.4%)		
Tracheal injury	311/703 (44.2%)	326/701 (46.5%)	1.0 (0.8–1.1)	0.421
Redness	225 (32.0%)	238 (34.0%)		
Edema	41 (5.8%)	34 (4.9%)		
Hematoma	27 (3.8%)	39 (5.6%)		
Bleeding	18 (2.6%)	15 (2.1%)		
Carina injury	91/703 (12.9%)	85/701 (12.1%)	1.1 (0.8–1.4)	0.687
Redness	39 (5.5%)	31 (4.4%)		
Edema	41 (5.8%)	37 (5.3%)		
Hematoma	11 (1.6%)	17 (2.4%)		
Left bronchial injury	539/703 (76.7%)	556/701 (79.3%)	1.0 (0.9–1.0)	0.246
Redness	249 (35.4%)	269 (38.4%)		
Edema	36 (5.1%)	47 (6.7%)		
Hematoma	201 (28.6%)	191 (27.2%)		
Bleeding	53 (7.5%)	49 (7.0%)		
Right bronchial injury	16/703 (2.3%)	27/701 (3.9%)	0.6 (0.3–1.1)	0.091
Redness	14 (2.0%)	24 (3.4%)		
Edema	2 (0.3%)	3 (0.4%)		
Sore throat 1 hour after surgery	268/715 (37.5%)	279/712 (39.2%)	1.0 (0.8–1.1)	0.514
Mild	178 (24.9%)	169 (23.7%)		
Moderate	75 (10.5%)	88 (12.4%)		
Severe	15 (2.1%)	22 (3.1%)		
Sore throat 1 day after surgery	290/715 (40.6%)	311/712 (43.7%)	0.9 (0.8–1.0)	0.239
Mild	191 (26.7%)	210 (29.5%)		
Moderate	86 (12.0%)	90 (12.6%)		
Severe	13 (1.8%)	11 (1.5%)		
Sore throat 2 day after surgery	157/715 (22.0%)	144/712 (20.2%)	1.1 (0.9–1.3)	0.437
Mild	111 (15.5%)	102 (14.3%)		
Moderate	42 (5.9%)	39 (5.5%)		
Severe	4 (0.6%)	3 (0.4%)		
Hoarseness 1 hour after surgery	187/715 (26.2%)	177/712 (24.9%)	1.1 (0.9–1.3)	0.585
Mild	107 (15.0%)	92 (12.9%)		
Moderate	75 (10.5%)	81 (11.4%)		
Severe	5 (0.7%)	4 (0.6%)		
Hoarseness 1 day after surgery	198/715 (27.7%)	186/712 (26.1%)	1.1 (0.9–1.3)	0.512
Mild	112 (15.7%)	106 (14.9%)		
Moderate	84 (11.7%)	76 (10.7%)		
Severe	2 (0.3%)	4 (0.6%)		
Hoarseness 2 day after surgery	134/715 (18.7%)	117/712 (16.4%)	1.1 (0.9–1.4)	0.266
Mild	87 (12.2%)	85 (11.9%)		
Moderate	43 (6.0%)	32 (4.5%)		

Values are mean ± standard deviation or number of patients (percentage). The intubation time were analysed using unpaired t-tests and the other outcomes were analysed using Fisher’s exact tests.

*Risk ratios of multiple intubation attempts.

P values were obtained by comparing one intubation attempt with two or three attempts.

**Fig 4:**
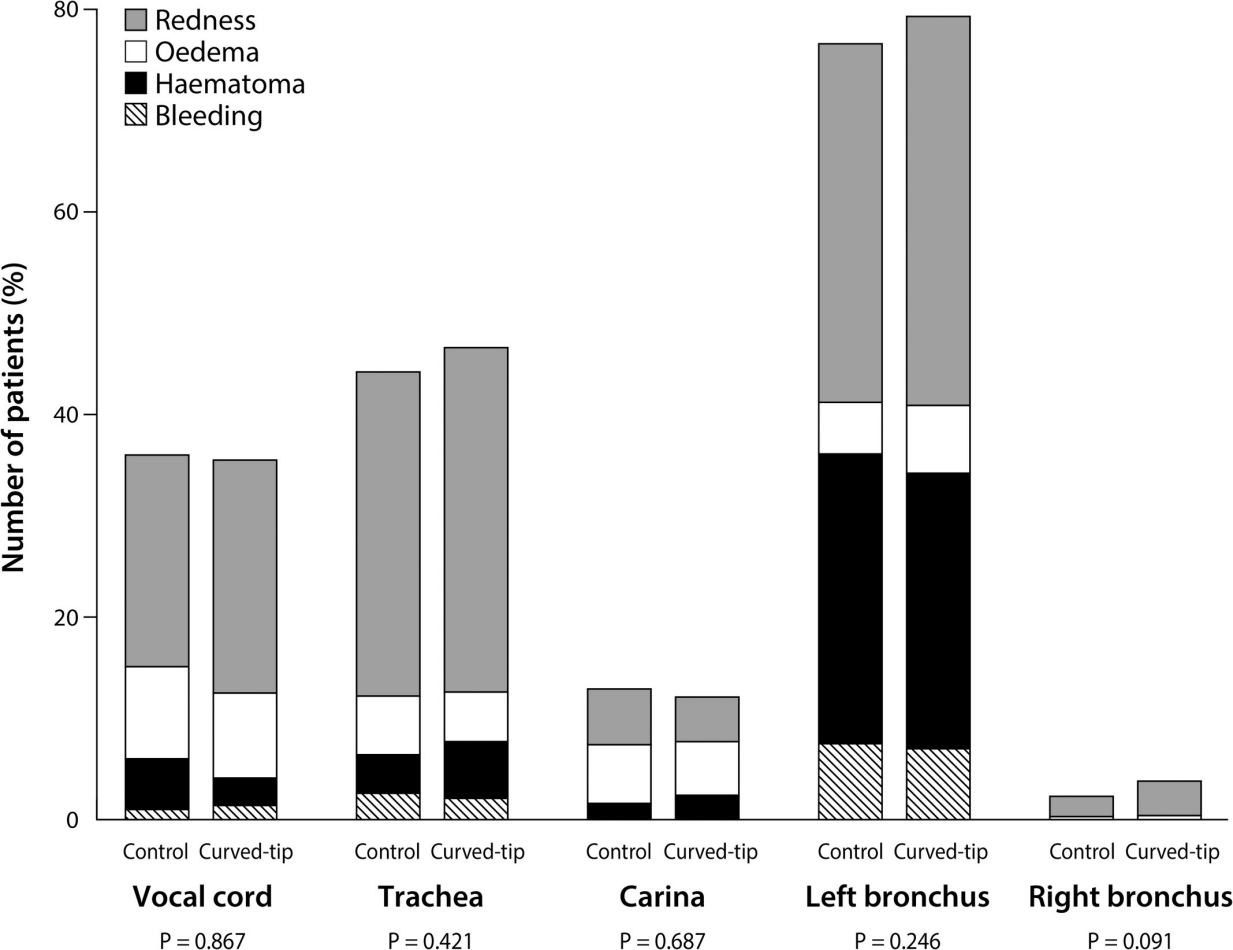
The site and type of postoperative airway injury. P values were obtained by comparing the incidence of airway injury between groups using Fisher’s exact tests.

**Fig 5:**
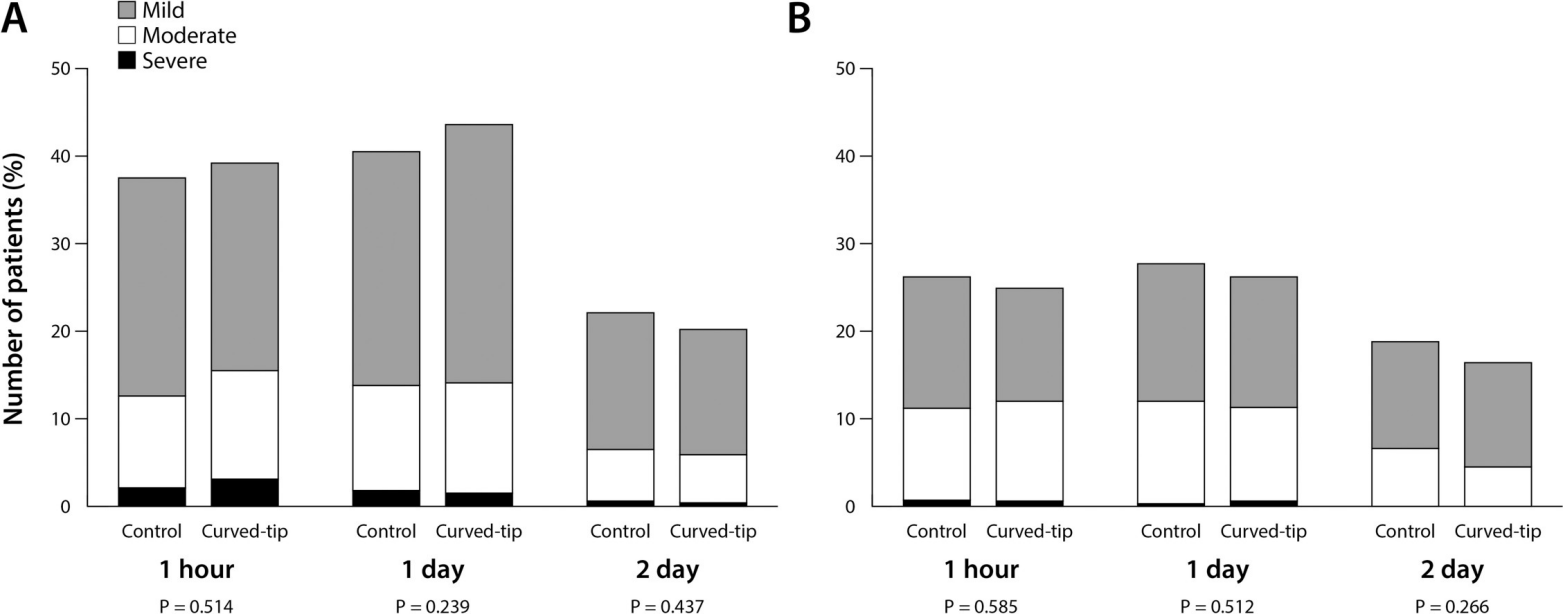
**The incidence and severity of sore throat (A) and hoarseness (B) one hour, one and two days after extubation of left-sided double-lumen tubes.** P values were obtained by comparing the incidence of sore throat (A) and hoarseness (B) between groups using Fisher’s exact tests.

Because the fiberoptic bronchoscopy was unavailable in 23 patients, airway injuries were examined in 1404 patients after surgery (Fig 1) and did not differ between groups (Fig 4, Table 4). No patients showed serious complications such as tracheobronchial laceration or perforation. The incidence and severity of sore throat and hoarseness were not different between groups until the second postoperative day (Fig 5, Table 4).

## Discussion

In our study, the left-sided DLT correctly entered the left mainstem bronchus more frequently in the curved-tip group than in the control group. Intubation was achieved more rapidly with fewer attempts in the curved-tip group. After extubation, signs or symptoms associated with airway injury did not differ between groups.

**Fig 6:**
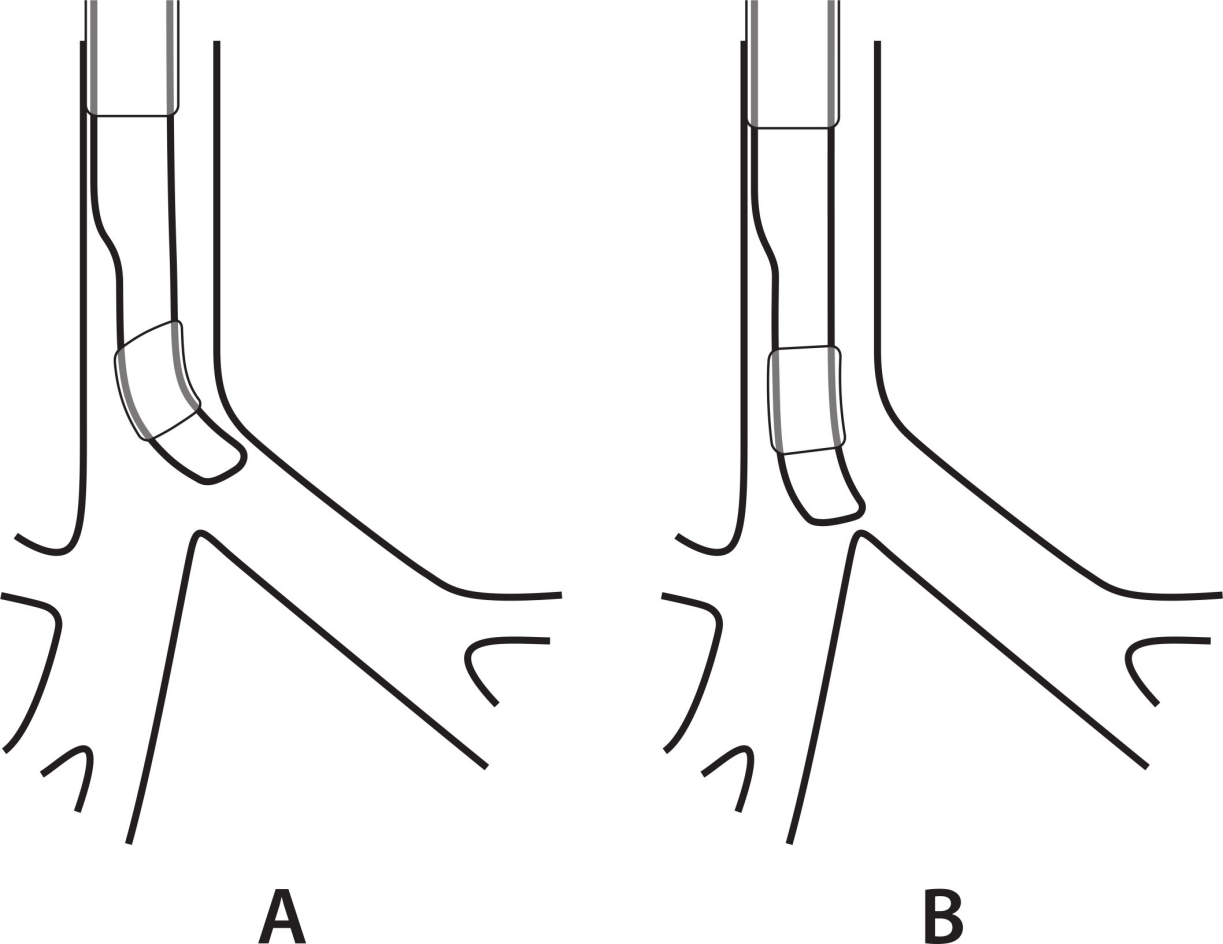
A diagram showing that the more curved tip of the left-sided double-lumen tube (A) is further extended to the left bronchial opening over the carina compared with the less curved tip (B).

A PVC DLT contains a plasticizer material such as dioctyl phthalate, so it is flexible and retains the transformed shape temporarily. [26] If the DLT tip is further bent and kept with a stylet inside before intubation, the more concave curvature can be maintained while it passes the trachea even after removal of the stylet. The more curved tip is further extended to the left bronchial opening over the carina (Fig 6). Therefore, the right bronchial misplacement seemed to be less frequent in the curved-tip group than in the control group.

Smaller DLTs are more likely to enter the right bronchus because the right bronchus is wider than the left bronchus. [2, 16, 27–29] Besides, The Mallinckrodt^TM^ 32-Fr DLT has more obtuse tip angle than the other sized tubes. [16] This may explain more frequent malpositioning in smaller DLTs and especially in the 32-Fr DLT.

Generally, it is not allowed to modify a clinical tool in a way which is different from the original version released by manufacturers. However, the stylet inside tracheal tubes is used to temporarily change the tube to a hockey-stick shape so as to improve access to the ‘*anterior larynx*’. [30–32] Similarly, the augmented curved tip of DLTs is more likely to be aligned with the glottis in the anterior direction and with the left bronchus in the left direction. This is a probable reason for the shorter time and fewer attempts for intubation in the curved-tip group, especially in the patients with the laryngoscopic grade of 2 or 3. Therefore, augmenting the curved tip of DLTs would be reasonable to facilitate tracheal intubation as well as to reduce the right bronchial misplacement of left-sided DLTs. In addition, deep neuromuscular and anesthetic levels should be ensured as in our study, because these factors play a major role in the difficulty of intubation. [33, 34]

Theoretically, up to 90°, the more concave tip can extend more laterally and thus align with the left bronchus (Fig 6). However, the stylet can be broken or hardly removed from the tube when it is too angulated. [30, 35, 36] Moreover, the excessively bent tip may intensify contact and damage to airway tissues during intubation. We thus bent the DLT tip only to an angle of 135° (= 180° - 45°) and observed fewer right bronchial misplacement without aggravating airway injury. However, our study did not show which angle of the DLT tip is most effective in reducing the right bronchial misplacement.

The practitioner’s proficiency may affect malpositioning of the DLT. [16, 37] In addition, the tip shape—group assignment—cannot be blinded to the practitioner of intubation. Therefore, instead of the investigators of our study, various anesthetists performed intubation and showed fewer right bronchial misplacement of left-sided DLTs in the curved-tip group than in the control group. It may not only extend the external validity but also minimize observer bias of our study.

This study has limitations. We only studied DLTs made of PVC, so our findings may not be extrapolatable to other materials such as silicone. Furthermore, we did not investigate the 41-Fr DLT because it seems too large for most Asians. [16] We would expect relatively few malpositions of the 41-Fr DLT based on the trend observed in Table 2.

## Conclusions

Before intubation of left-sided DLTs, augmentation of the curved DLT tip can reduce the right bronchial misplacement and facilitate intubation without aggravating airway injury.

## Supporting information

S1 Checklist CONSORT checklist. (DOCX)

S1 Protocol Study protocol (Korean). (DOCX)

S2 Protocol Study protocol (English). (DOCX)

S1 Dataset Dataset of the study. (XLSX)
