# Supplementary material for: Augmentation of curved tip of left-sided double-lumen tubes to reduce right bronchial misplacement: A randomized controlled trial
Source: PLoS One. 2019 Jan 15;14(1):e0210711. doi: 10.1371/journal.pone.0210711 (PMC6333363; doi:10.1371/journal.pone.0210711)
Supplement: S1 Protocol — (DOCX) [file pone.0210711.s002.docx]

# **연구계획서**

| 좌측 이중관 기관지튜브가 우측 주기관지로 잘못 거치되는 것을 막기 위한 전처치 방법 |
| --- |

일시

2013년 IRB 통과시점 – IRB 통과 후 2년

서울대학교 의과대학 마취통증의학과 교실

교수 박 재 현

**1. 연구 제목**

좌측 이중관 기관지튜브가 우측 주기관지로 잘못 거치되는 것을 막기 위한 전처치 방법

(영어제목: The pretreatment of a left-sided double lumen tube to prevent its misplacement to the right mainstem bronchus)

**2. 연구의 실시기관명 및 주소**

서울대학교 의과대학 마취통증의학과 교실, 서울특별시 종로구 대학로 101

**3. 연구의 책임자 및 담당자**

**3.1. 연구책임자**

박재현

교수

서울특별시 종로구 대학로 101, 서울대학교 마취통증의학과

전화번호: 02-2072-2818

**3.2. 연구담당자**

서정화

임상조교수

서울특별시 종로구 대학로 101, 서울대학교 마취통증의학과

전화번호: 02-2072-7361

**4. 연구 배경 및 목적**

**4.1. 연구 배경**

일측 폐환기를 시행할 때 흔히 사용하는 좌측 이중관 기관지튜브(left double-lumen endobronchial tube, DLT)의 삽관 시 직접후두경을 이용하여 DLT의 bronchial tip이 glottis를 통과할 때까지 삽관 후 좌측으로 90° 회전시키고 전진시켜 DLT의 bronchial lumen의 원위부가 좌측 주기관지(left mainstem bronchus, LMB) 내에 거치되게 한다. 그러나 LMB에 비해 우측 주기관지(right mainstem bronchus, RMB)가 trachea와 이루는 각도가 더 크기 때문에 단일관 기관튜브(single lumen endotracheal tube)를 깊게 삽관하면 대부분 RMB로 삽관되는 것처럼 DLT의 경우에도 정확한 방법으로 삽관했음에도 불구하고 RMB로 잘못 들어갈 수 있다. 이러한 경우 DLT이 LMB로 들어가도록 다시 위치 교정을 해야 하는데 이 과정에서 저산소증, 고탄산혈증, 기도 손상 등 합병증이 생길 수 있기 때문에 DLT를 한번에 LMB에 정확하게 거치시키는 것은 일측 폐환기를 시행함에 있어 매우 중요한 부분이라 할 수 있다. 이전 연구에서 DLT에 stylet을 넣어둔 채로 삽관을 하면 DLT의 형태가 유지되어 DLT이 LMB로 정확하게 들어가는 빈도가 높아진다고 보고하였으나 이는 기도 손상의 위험성을 높힐 수 있기 때문에 일반적으로 임상에 적용하기는 힘들 것이라 생각된다.

현재 본원에서 사용되는 DLT (Mallinckrodt^TM^ Endobronchial tube, Covidien, Dublin, Ireland)는 polyvinyl chloride에 가소제 dioctyl phthalate가 함유되어 있는 재질로 flexibility와 rigidity를 함께 가지고 있고 원래의 모양에서 일정 시간 힘을 가하여 모양을 변형시키면 수분간 변형된 모양을 유지하다가 서서히 원래 모양으로 돌아오는 특징을 가지고 있다. 실제로 32 Fr DLT에 stylet을 넣어둔 채로 bronchial tip을 45°로 꺾고 5분 간 유지한 후 stylet을 제거했더니, stylet 제거 직후 bronchial tip의 각도가 35°였고 시간이 지날수록 각도가 서서히 줄어들다가 약 10분 후에 원래 DLT의 형태로 돌아가는 걸 확인할 수 있었다.

이렇게 DLT의 bronchial tip이 꺽인 채로 유지된다면 DLT 삽관 시 튜브가 RMB로 잘못 들어갈 위험이 낮아질 것으로 예상되어 이 실험을 계획하게 되었다.

**4.2. 연구목적 및 가설**

기관내 삽관 전 DLT에 stylet을 넣어둔 채로 45°로 구부리고 5분간 유지했을 때 DLT이 RMB로 잘못 들어갈 위험을 낮출 수 있는지 알아보고자 한다. 가설은 “기관내 삽관 전 DLT에 stylet을 넣어둔 채로 45°로 구부린 경우, 그러지 않은 경우에 비해 DLT가 RMB로 잘못 들어가는 빈도가 더 낮을 것이다”이다.

**5. 대상질환 및 대상자**

좌측 DLT를 삽관하여 흉부외과 수술을 받는 환자

**6. 예상 연구기간**

2013년 IRB 통과시점 이후 2년

**7. 피험자 선정 및 피험자 수**

**7.1. 피험자의 선정 기준, 제외 기준**

**선정기준**

좌측 DLT를 삽관하여 흉부외과 수술을 받는 20세 이상 85세 이하 연령의 환자

**제외기준**

시험에 동의하지 않는 환자

수술전 영상 검사 상 tracheobronchial tree에 anatomy가 있는 환자

LMB, RMB에 intraluminal lesion이 있는 환자

Difficult intubation이 예상되는 환자

**중도탈락 및 임상시험 중지 기준**

피험자의 동의 철회

DLT 삽관을 시도했으나 실패한 경우

**7.2 피험자 수**

DLT에 아무런 형태 변화를 가하지 않은 상태로 삽관하는 군(대조군): 770명

삽관 전 DLT에 stylet을 넣어둔 채로 bronchial tip을 45°로 구부려서 5분간 유지한 후 삽관하는 군(시험군): 770명

**8. 임상시험 일정**

2013년 IRB 통과 직후 – IRB 통과 후 2년

2013년 IRB 통과 직후 1개월: 시험 준비

2013년 IRB 통과 1개월 후부터 23개월: 임상시험

2013년 IRB 통과 23개월 후부터 1개월: 자료 정리 및 논문 작성

**9. 연구 방법**

**9.1. 대상자 선정**

좌측 DLT를 삽관하여 흉부외과 수술을 받는 환자들 중 상기된 선정, 제외기준에 부합되고 환자가 동의할 경우 대상자로 선정한다.

**9.2. 피험자 동의확보**

별첨된 설명문과 동의서에 따라 연구 담당자, 공동 연구자가 피험자에게 설명한 후 서면 동의를 받는다.

**9.3. 무작위 배정**

대조군: DLT에 아무런 형태 변화를 가하지 않은 상태로 삽관하는 군

시험군: 삽관 전 DLT에 stylet을 넣어둔 채로 bronchial tip을 45°로 구부려서 5분간 유지한 후 삽관하는 군

피험자 등록 전 DLT의 삽관에 관여하지 않는 의사가 대조군(A)과 시험군(B)을 무작위 난수표에 따라 무작위로 배정하고, 이 배정표에 따른 순서대로 피험자를 대조군이나 시험군에 배정하여 시험을 진행한다.

**9.4. 목표 피험자의 수 및 산출 근거**

이전 연구(unpublished, IRB No. H-1105-027-360)에서 DLT가 우측으로 잘못 거치된 경우가 4.2%였다. 이번 연구의 시험군에서 대조군에 비해 DLT가 우측으로 잘못 거치되는 빈도를 60% 이상 감소시키면(4.2% → 1.68%) 임상적으로 유의하다고 보고 0.05의 알파 값과 0.8의 power를 가정하였을 때 각 군당 702명의 피험자가 필요한 것으로 계산되었다. 약 10%의 탈락율을 고려하여 각 군당 770명의 피험자 모집을 목표로 하였다.

**9.5. 임상 시험의 시행계획**

Intubation 전 DLT의 bronchial tip을 미리 구부리는 여부를 제외한 모든 임상 처치는 두 군에서 동일하게 시행한다.

마취의 유도는 propofol과 remifentanil을 이용한 총정맥마취(total intravenous anesthesia)로 시행한다. 두 약제의 효과처 농도(effect site concentration)를 propofol 3-5 mcg/ml, remifentanil 3-5 ng/ml로 설정하여 약제 주입을 시작하고 피험자의 의식이 소실되면 용수 환기를 하면서 rocuronium 0.6-0.8 mg/kg을 투여한다. 3분 후 직접후두경을 이용하여 DLT의 bronchial tip이 glottis를 통과할 때까지 intubation 후 stylet을 제거하고 DLT를 반시계 방향으로 90° 회전시킨 후 적절한 깊이까지 DLT를 전진시킨다. 대조군은 DLT의 모양을 변형시키지 않은 상태로, 시험군은 bronchial tip을 45° 구부리고 5분간 유지시킨 후 intubation을 시행한다. Intubation은 DLT의 intubation에 능숙한 마취통증의학과 전문의 지도 하에 마취통증의학과 전공의가 1차적으로 시행하고 실패 시 지도하는 전문의가 시행한다. Intubation 후 fiberoptic bronchoscope (FOB; LF-DP or LF-GP, Olympus Optical Co., Tokyo, Japan)을 이용하여 DLT가 LMB로 정확하게 들어갔는지 유무를 확인하고 정확한 위치를 조정한다.

수술이 끝나고 양측 폐환기를 시행하는 시점에 DLT를 proximal trachea까지 뺀 후 DLT를 통해 FOB를 삽입하여 LMB, RMB, trachea, carina의 손상(redness, edema, hematoma, bleeding 등) 유무를 관찰한다. Extubation 후 vocal cord injury를 관찰한다.

수술 1시간, 1일, 2일 후에 sore throat, hoarseness를 평가하여 none, mild, moderate, severe로 분류한다.

**9.6. 관찰항목, 검사항목**

**관찰항목**

성별, 나이, 신장 체중, DLT size, Cormack and Lehane’s grade, intubation 시도 횟수, intubation에 소요된 시간, LMB로의 삽관 여부, 수술체위(좌측와위/우측와위/앙와위/기타), 수술 형태 (VATS/thoracotomy/sternotomy/기타), 수술 시간, 마취 시간 등을 기록한다.

**검사항목**

Extubation 전에 FOB으로 airway injury의 위치, 형태 등을 평가한다.

병변의 위치: LMB, RMB, trachea

병변의 형태: redness, edema, hematoma 등

**9.7. 판정 기준**

DLT intubation 후 FOB을 통해 DLT가 LMB로 정확하게 들어갔는지 확인하고, extubation 전 FOB을 통해 양쪽 main bronchus와 trachea를 관찰하여 손상의 위치, 형태 등을 평가한다.

**9.8. 안전성 평가**

본 시험에 참여한 모든 피험자를 대상으로 안전성 평가를 실시한다.

연구 시행 중에 이상반응이 발생했을 경우, 연구에 사용된 시험 방법과의 인과 관계 유무와 모든 이상 반응을 기록하고 추후 중증도, 중대성, 기간, 그리고 시험 방법과의 인과관계를 평가한다. 이상반응에 대한 처치 및 결과 역시 기록한다. 이상 반응은 시험 기간 중의 계획된 검진과 검사의 소견 이외에도 비정상적인 검진이나 필요에 따른 추가적인 검사와 검진에 의해 평가하고 즉각적인 조치를 시행한다. 임상병리검사 자료에 대해서는 변수의 특성에 따라 치료 전, 후의 군내 비교 등 적절한 통계적 방법을 이용하여 분석하고, 이상반응의 빈도, 발현율, 각각의 목록, 심각한 정도 및 시험 방법과의 인과관계 등을 제시하며, 필요한 경우 그래프 형태로 보고한다.

1)중증도

이상반응은 아래의 정의에 따라 경증, 중등증, 중증으로 구분된다.

경증은 일반적이고 일시적이고 일상적인 활동을 방해하지 않는다.

중등증은 약간의 불편함을 초래하거나 일상적인 활동을 방해한다.

중증은 일상적인 활동을 수행할 수 없다.

2)인과 관계

인과 관계는 ‘관련 없을 것으로 생각됨’, ‘관련 있을 가능성 있음’, ’가능성 많음’, ’명백히 관련 있음’, 또는 ‘관련성을 확인하기 어려움’으로 구분된다.

3)이상 반응의 분류

**DLT의 삽관과 관련된 이상 반응**

DLT의 삽관 시 수술 후 hoarseness, sore throat 증상이 발생할 수 있으나 3일 이상 지속되지는 않으며, vocal cord, trachea 등 airway injury가 생길 수 있으나 redness, edema 등의 경미한 합병증이 대부분인 것으로 보고되고 있다.

**DLT의 끝을 구부리는 전처치와 관련된 이상 반응**

DLT은 single-lumen tube에 비해 외경이 크고 주기관지까지 깊숙히 삽관해야 하기 때문에 삽관 과정에서 기도 손상이 더 잘 생기는 것으로 알려져 있다. DLT의 끝을 구부리는 전처치를 할 경우 DLT와 기도의 접촉이 증가하여 기도 손상을 더 유발할 가능성이 있다.

**수술 후 추가적인 bronchoscopy와 관련된 이상 반응**

DLT 삽입 후 시행하는 bronchoscopy는 튜브 위치를 확인하기 위한 것으로 일반적인 시술이나 수술 후 추가적인 bronchoscopy는 이 연구에서 airway injury를 평가하기 위해 시행하는 것으로 일반적인 시술은 아니다. Bronchoscopy 자체가 기도 손상을 더 유발할 가능성이 있다.

**9.9. 통계 분석 원칙 및 방법**

무작위배정 후 중도 탈락된 경우를 제외한 모든 피험자를 대상으로 통계 분석을 시행한다. 대조군과 시험군의 성별, DLT size, Cormack and Lehane’s grade, intubation 시도 횟수, 수술 체위, 수술 형태, airway injury의 위치, 형태 등 범주형 변수는 Fisher’s exact test를 이용하여 비교 분석한다. 대조군과 시험군의 나이, 신장, 체중, intubation에 소요된 시간, 수술 시간, 마취 시간 등 연속형 변수는 unpaired or paired *t*-test 혹은 Mann-Whitney U test or Wilcoxon signed-rank test를 이용하여 비교 분석한다. *P* 값이 <0.05인 경우 통계학적으로 유의한 것으로 판단한다.

**10. 피험자 안전보호에 관한 대책**

DLT의 삽관 시 수술 후 일시적인 hoarseness, sore throat 증상이 발생할 수 있으나 3일 이상 지속되지는 않으며, vocal cord, trachea 등 airway injury가 생길 수 있으나 경미한 합병증이 대부분인 것으로 보고되고 있다. DLT의 끝을 구부리는 전처치를 하면 삽관 과정에서 DLT와 기도의 접촉이 증가하여 기도 손상을 더 유발할 가능성이 있지만 stylet 이 제거된 상태에서의 DLT는 다소 유연한 상태이기 때문에 DLT 끝의 상태가 단지 더 구부러진 것만으로 기도 손상이 더 심해지지는 않을 것이라 생각한다. 그리고 bronchoscopy 자체가 기도 손상을 더 유발할 수 있으나 이 시험에서의 bronchoscopy는 이미 삽관되어 있는 튜브를 통해 시행하고, 수술 전 진단 목적으로 시행하는 일반적인 FOB에 비해 내경이 상당히 작은(3-4 mm) FOB을 이용하며, FOB에 능숙한 마취통증의학과 전문의가 직접 시행하므로 bronchoscpy와 관련된 추가 위험의 가능성은 작을 것이라 생각된다.

**11. 연구의 윤리성 확보를 위한 방안**

2008 헬싱키 선언에 입각하여, 피험자 또는 보호자에게 연구의 목적과 연구 참여 중 일어날 수 있는 정신적, 신체적 위해를 충분히 설명한 후 피험자 (또는 보호자) 로부터 서면동의서를 받을 예정이다. 피험자, 피험자의 담당의사, 시험 참여자 이외에는 피험자의 시험 참여 여부나 치료 경과에 대해 알지 못하게 하며, 피험자의 신원을 파악할 수 있는 기록은 비밀로 보장될 것이다. 연구를 위해 수집되는 정보는 잠금장치가 있는 연구실에 비밀번호가 걸린 파일로 보관하고 연구파일에 접근할 수 있는 사람은 권한을 가진 일부 연구원으로 제한할 것이다. 수집되는 자료의 불필요한 개인식별자는 제거하고, 특히, 증례기록서에는 피험자의 이름, 주민등록번호, 차트 번호 등을 기재하지 않도록 하며, 신상정보와 연결된 식별자 코드는 별도로 관리할 것이다. 피험자와 관련된 사진을 제출할 때는 피험자의 신원을 알 수 없도록 할 것이며 조금이라도 신원이 노출될 가능성이 있는 경우에는 이에 대한 서면 동의를 받았음을 명시할 것이다. 피험자의 검진 기록은 비밀이 유지되고 다른 곳으로 이동되지 않을 것이며, 본 연구의 진행 여부를 감독 받기 위해 감독 기관으로 보내어질 수 있다. 그리고 이 자료는 법이 정하는 기간 동안 보관될 것이며 추후 모든 자료는 폐기될 것이다. 박성원 간호사가 정기적으로 시험기관을 방문하여 피험자 모집, 피험자 등록, 데이터 저장 및 분석에 대한 평가, 임상시험계획서와 GCP의 준수 여부를 확인할 것이다. 본 연구는 병원윤리위원회의 윤리규정을 준수할 것이다.

**12. 참고문헌**

1. Neustein SM, Eisenkraft JB. Proper lateralization of left-sided double-lumen tubes. Anesthesiology. 1989 Dec;71(6):996.

2. Lieberman D, Littleford J, Horan T, Unruh H. Placement of left double-lumen endobronchial tubes with or without a stylet. Can J Anaesth. 1996 Mar 1;43(3):238–42.

3. Knoll H, Ziegeler S, Schreiber J-U, et al. Airway injuries after one-lung ventilation: a comparison between double-lumen tube and endobronchial blocker: a randomized, prospective, controlled trial. Anesthesiology. 2006 Sep;105(3):471–7.
