# Supplementary material for: Augmentation of curved tip of left-sided double-lumen tubes to reduce right bronchial misplacement: A randomized controlled trial
Source: PLoS One. 2019 Jan 15;14(1):e0210711. doi: 10.1371/journal.pone.0210711 (PMC6333363; doi:10.1371/journal.pone.0210711)
Supplement: S2 Protocol — (DOCX) [file pone.0210711.s003.docx]

**Clinical Study Protocol**

**(Version 1.0)**

**1. Study Title**

The pretreatment of a left-sided double-lumen tube to prevent its misplacement to the right mainstem bronchus

**2. Address of Research Center**

Department of Anesthesiology and Pain Medicine, Seoul National University Hospital, Seoul National University College of Medicine, 101 Daehak-ro, Jongno-gu, Seoul 03080, Korea

**3. Principal investigators and Co-investigators**

3.1 Principal Investigators

Jae-Hyon Bahk, Professor

Department of Anesthesiology and Pain Medicine, Seoul National University Hospital, Seoul National University College of Medicine

3.2 Co-investigator

Jeong-Hwa Seo, Clinical professor

Department of Anesthesiology and Pain Medicine, Seoul National University Hospital, Seoul National University College of Medicine

**4. Responsibility of trial**

Investigator-initiated trial

**5. Address of sponsorship**

None

**6. Study period**

24 months after the approval of IRB

**7. Subjects of study**

Patients undergoing elective surgery with left-sided double-lumen tubes

**8. Necessity of Study and Overview**

8.1 Background

For one-lung ventilation during thoracic surgery, a left-sided double-lumen tube (DLT) can be correctly placed into the left mainstem bronchus by a blind method or by fiberoptic bronchoscopic guidance. With the blind method, the DLT is advanced into the left mainstem bronchus by turning its curved tip of the bronchial lumen to the left side. However, the DLT can be misplaced into the right mainstem bronchus because the right bronchus is wider and more vertical than the left bronchus. This right bronchial misplacement inhibits ventilation or collapse of the right upper lobe worsening one-lung ventilation and oxygenation.

In our previous study, we found that the DLT with an obtuse tip angle was more likely to enter the right bronchus. Therefore, we hypothesize that the right bronchial intubation of left-sided DLTs would decrease with the more acute tip angle. To prove this hypothesis, we plan a prospective randomized trial comparing the right misplacement of left-sided DLTs with different tip angles.

8.2 Hypothesis and objectives

The hypothesis is that the right bronchial misplacement of left-sided DLTs decreases with the more acute tip angle of the DLT compared to the more obtuse angle.

**9. Study materials**

PVC left-sided double-lumen tube (Mallinckrodt endobronchial tube; Covidien, Mansfield, MA, USA)

**10. Inclusion and exclusion criteria**

10.1 Inclusion criteria

Patients aged 20-85 years with ASA physical status of I-III who receive elective surgery with left-sided DLTs

10.2 Exclusion criteria

Patients with abnormal anatomy of tracheobronchial tree or clinical predictors of difficult intubation

10.3 Sample size calculation

In our previous study, the incidence of the right bronchial misplacement of left-sided DLTs was 4.2%. When considering a 60% decrease of the incidence in the curved-tip group compared with the control group, 705 patients were required in each group with a type-I error risk of 0.05 and a power of 0.8 for two-sided analysis.

10.4 How to recruit

Potential participants who meet the inclusion and exclusion criteria (See 10.1 and 10.2) are recruited at outpatient clinics or on the preoperative visit before surgery, and written informed consents are obtained from all of the participants.

**11. Study protocols**

11.1 Interventions

Before surgery, we measure the inner diameters of the left and right mainstem bronchi on the preoperative computed tomography. The DLT size is chosen based on the left bronchial diameter or sex and height of the patient. Nurses not involved in the study protocol prepare the DLT. The DLT has a stylet inside the bronchial lumen, and the tracheal and bronchial cuffs are deflated. At the midpoint between the proximal margin of the bronchial cuff and the radiopaque line, the curved tip is further bent to an angle of 135° in the curved-tip group, but not in the control group.

Anesthesia is induced with effect-site target-controlled infusion of propofol and remifentanil. Rocuronium 0.6–0.8 mg kg^-1^ is administered and train-of-four (TOF) counts are monitored. At a TOF count=0 and bispectral index of <60, intubation is performed under direct laryngoscopy with a Macintosh blade of 3 or 4. The bronchial tip is inserted into the glottis directing anteriorly. The DLT is rotated 90° counterclockwise turning the tip towards the left and advanced to the bronchus, and then the laryngoscopic blade is removed from the mouth.

Using a fiberoptic bronchoscope, we check which bronchus is intubated. If the DLT enters the right mainstem bronchus, it is withdrawn back to the trachea and it is guided into the left bronchus with the bronchoscope. It is re-checked after positional change of the patient.

The patient is turned to the supine position after surgery. A blinded investigator examines the vocal cords, trachea, carina, both bronchi using a fiberoptic bronchoscope. The types of injuries are categorized as redness, edema, hematoma, bleeding, and others.

A blinded investigator evaluates sore throat and hoarseness one hour, one and two days after extubation and grades as none, mild, moderate, and severe symptoms.

11.2 Randomization and Blinding

Patients are randomized into two groups depending on whether the DLT tip is further bent or not before intubation. Group assignment is randomized in a 1:1 ratio by a clinician not involved in the study Patients and investigators are blinded to group assignment.

11.3 Administration and dose of drug

N/A

11.4 Outcome measurements

- Baseline data: sex, age, body weight, height, body mass index, surgical position, type of surgery, duration of surgery and anesthesia

- Cormack and Lehane’s grade

- Number of attempts for intubation

- intubation time

- Intubated bronchus (left or right)

- Site and type of postoperative airway injury

- Incidence and severity of postoperative sore throat and hoarseness

11.5 Primary outcome measurements

Incidence of right bronchial intubation of left-sided DLTs

11.6 Management of adverse events

All interventions are monitored by trained anesthesiologists and if complications occur, appropriate treatments are provided to decrease additional risk to the patient.

11.7 Criteria for Discontinuation and Drop-out

Withdrawal of consent

11.8 Plan for report of harmful cases

Reports of serious adverse events (airway-related problems such as hypoxic brain damage, hypoxic organ damage, etc.) and unexpected problems and research-related adverse events are reported to the IRB within 7 days, and within 24 hours for serious and unexpected adverse events.

11.9 Data Safety Monitoring Plan

Principal investigator will make comparisons between the source documents and the study protocol at every 6 months to assure the completeness of data and will review the safety data of subjects. The subject identification code on the data will be encoded, and will be filed in a safe installed with a locking device while the electronic document will be saved in a computer which is restricted for access.

**12. Statistical analysis**

Continuous variables are summarised as mean (standard deviation) or median (interquartile range) and analyzed with unpaired or paired t-tests and Mann-Whitney U or Wilcoxon signed-rank tests. Categorical variables are presented as the number of patients (%) and compared with Fisher’s exact test. Effect sizes with 95% confidence intervals are calculated. A significance criterion is P = 0.05 and adjusted with Bonferroni correction as appropriate

**13. References**

1. Neustein SM, Eisenkraft JB. Proper lateralization of left-sided double-lumen tubes. Anesthesiology. 1989 Dec;71(6):996

2. Lieberman D, Littleford J, Horan T, Unruh H. Placement of left double-lumen endobronchial tubes with or without a stylet. Can J Anaesth. 1996 Mar 1;43(3):238–42

3. Knoll H, Ziegeler S, Schreiber J-U, et al. Airway injuries after one-lung ventilation: a comparison between double-lumen tube and endobronchial blocker: a randomized, prospective, controlled trial. Anesthesiology. 2006 Sep;105(3):471–7
